# Supplementary material for: Dupilumab-associated head and neck dermatitis shows a pronounced type 22 immune signature mediated by oligoclonally expanded T cells
Source: Nat Commun. 2024 Apr 2;15:2839. doi: 10.1038/s41467-024-46540-0 (PMC10987549; doi:10.1038/s41467-024-46540-0)
Supplement: Supplementary file 1 — Supplementary Information [file 41467_2024_46540_MOESM1_ESM.pdf]

SUPPLEMENTARY INFORMATION

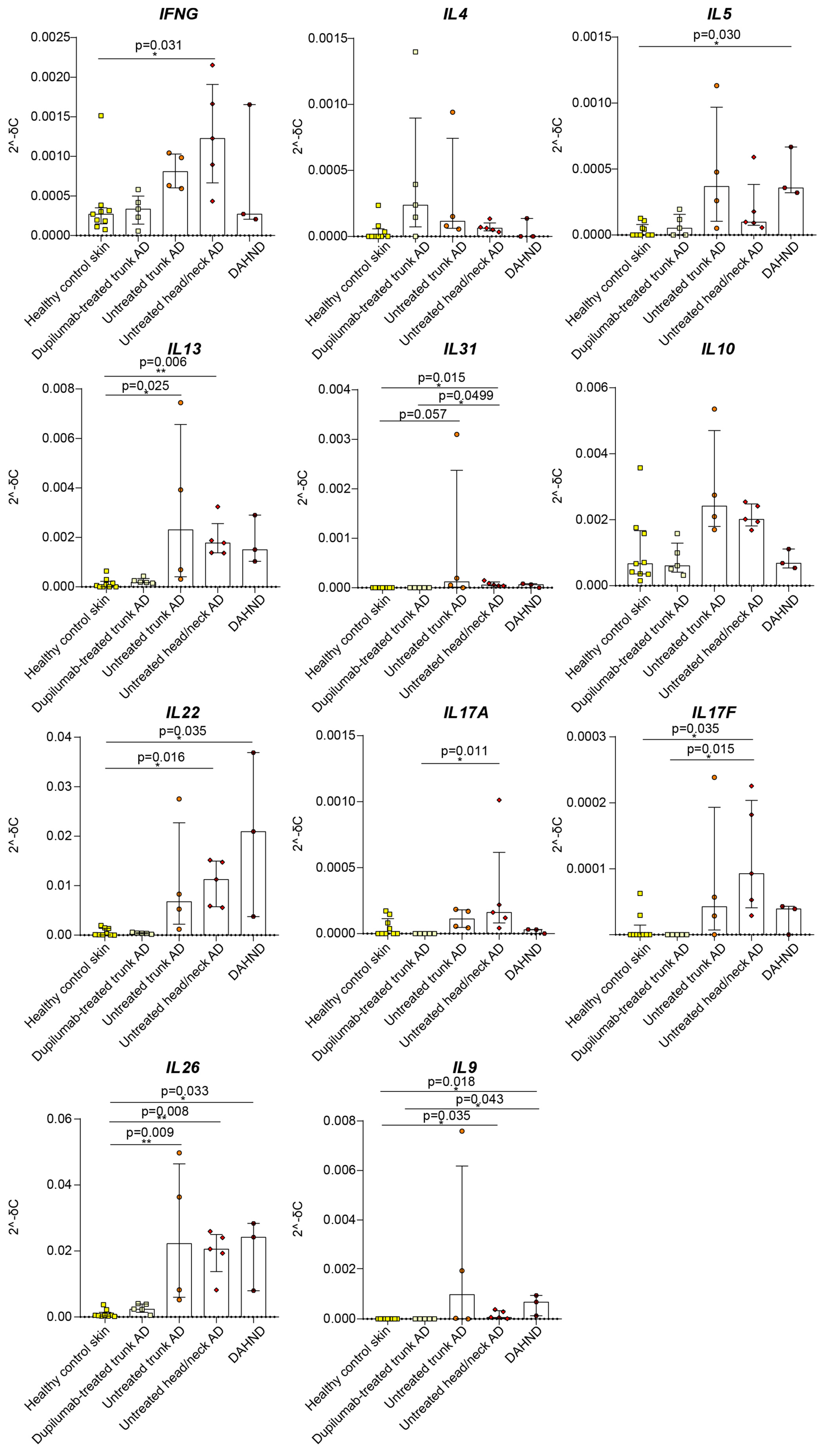

Figure S1. Quantitative RT-PCR of key T-cell cytokines. Healthy control skin (HC), dupilumab-treated trunk AD (n=5), untreated trunk AD (n=4), untreated head/neck AD (n=5), dupilumab-associated head and neck dermatitis (n=3); each dot represents a single donor. Statistical significance was calculated using a Kruskal-Wallis test for multiple comparisons followed by Dunn's post-hoc test.

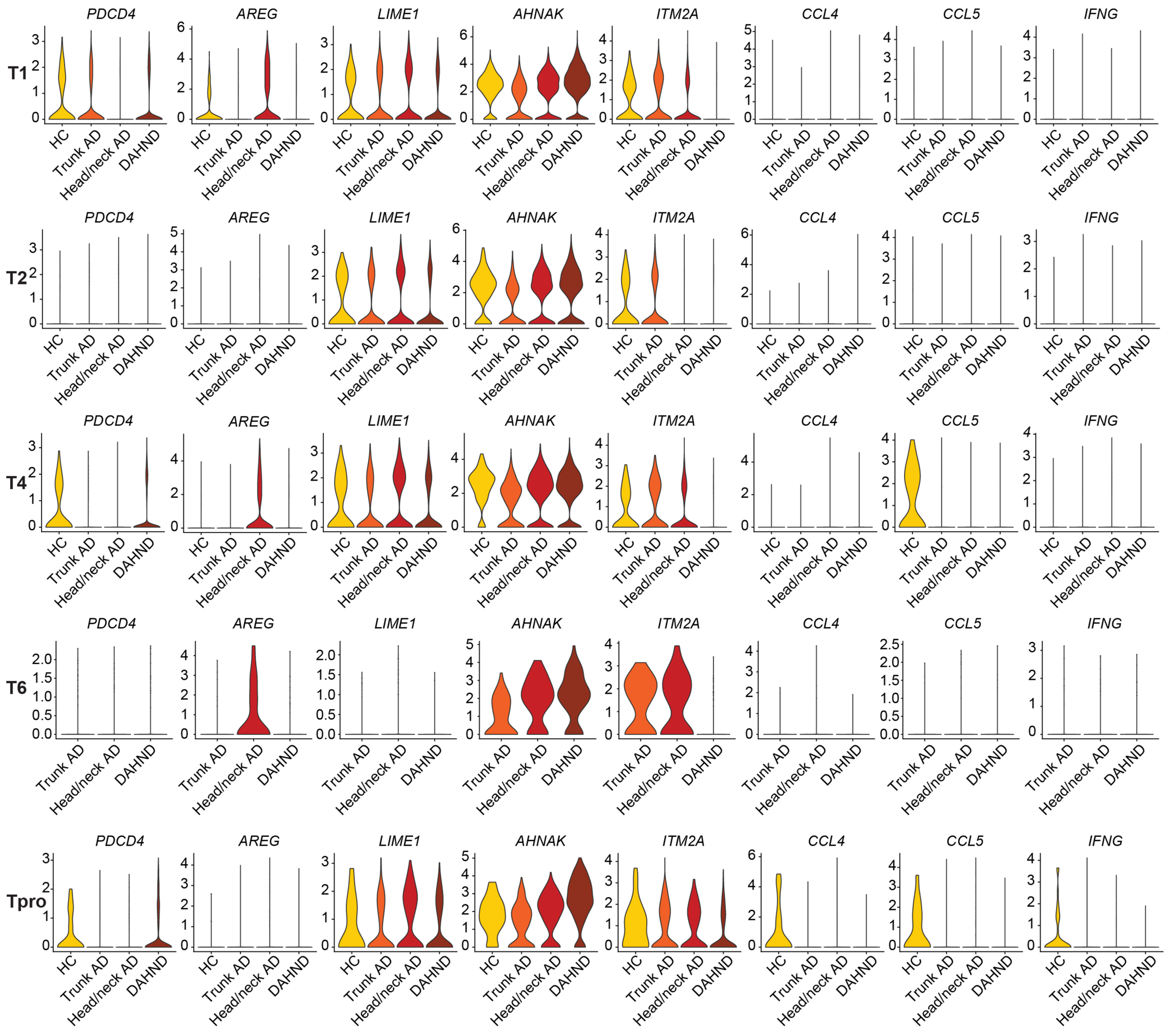

Figure S2. Violin plots of selected genes in T1, T2, T4, T6 and Tpro lymphoid clusters. Y-axis indicates expression levels.
