# Supplementary material for: Dupilumab-associated head and neck dermatitis shows a pronounced type 22 immune signature mediated by oligoclonally expanded T cells
Source: Nat Commun. 2024 Apr 2;15:2839. doi: 10.1038/s41467-024-46540-0 (PMC10987549; doi:10.1038/s41467-024-46540-0)
Supplement: Supplementary file 3 — Description of Additional Supplementary Files [file 41467_2024_46540_MOESM3_ESM.pdf]

Description of Additional Supplementary Files

Supplementary Data 1. Description: Patient baseline characteristics at time of sampling; MTX methotrexate; PUVA psoralen ultraviolet A; NB-UVB narrow-band ultraviolet B; TCS topical corticosteroids; TCI topical calcineurin inhibitors; IVIG intravenous immunoglobulins; CsA cyclosporine A; DAHND dupilumab-associated head and neck dermatitis; AD atopic dermatitis; HC healthy control skin; scRNA-seq single-cell RNA sequencing; IF immunofluorescence; n.a. not applicable; eow every other week. *In DAHND patients, the depicted IgE levels were measured before initiation of dupilumab treatment; ** IGA Investigator Global Assessment Score: In DAHND patients, facial lesions were not included in the IGA score. *** Topical treatments (TCI or TCS) were not applied at least 4 days before obtaining the skin biopsy.

Supplementary Data 2. Cell numbers per patient and cell type after quality control (QC) filtering and UMAP clustering.

Supplementary Data 3. Markers of individual cell clusters. Top 10 differentially expressed genes for each cluster ordered by $\log_2$ fold change and sorted by the adjusted p-value<0.05 using a two-sided Wilcoxon Rank Sum test with Bonferroni correction.

Supplementary Data 4. Differential gene expression for each cell cluster between HC, untreated trunk AD, untreated head/neck AD, and DAHND samples. Differential gene expression was defined as $\log_2$ fold change >│ ± 0.25│ and adjusted p<0.05 using a two-sided Wilcoxon Rank Sum test and Bonferroni correction.

Supplementary Data 5. Differential gene expression for each cell cluster between untreated head/neck AD and DAHND samples. Differential gene expression was defined as $\log_2$ fold change >│ ± 0.25│ and adjusted p<0.05 using a two-sided Wilcoxon Rank Sum test and Bonferroni correction.

Supplementary Data 6. Differentially expressed genes (DEGs) of top 10 expanded clones of DAHND, head/neck AD and trunk AD with their respective polyclonal TCR+ population, and their overlap between groups. Differential gene expression was defined as log2 fold change >│ ± 0.25│ and adjusted p<0.05 using a two-sided Wilcoxon Rank Sum test with Bonferroni correction.

Supplementary Data 7. Differentially expressed genes (DEGs) of top 10 expanded clones comparing trunk AD, head/neck AD and DAHND. Differential gene expression was defined as log2 fold change >│ ± 0.25│ and adjusted p<0.05 using a two-sided Wilcoxon Rank Sum test with Bonferroni correction.

Supplementary Data 8. Cells from the whole dataset were compared between four conditions (HC, trunk AD, head/neck AD and DAHND) in a pseudo-bulk analysis, and differentially expressed genes were selected with an adjusted p value <0.05, a log fold change >0.25 and min.pct value of 0.001 using a two-sided Wilcoxon Rank Sum test with Bonferroni correction.

Supplementary Software. The code for the data generated in this study.
