# Supplementary material for: Dupilumab-associated head and neck dermatitis shows a pronounced type 22 immune signature mediated by oligoclonally expanded T cells
Source: Nat Commun. 2024 Apr 2;15:2839. doi: 10.1038/s41467-024-46540-0 (PMC10987549; doi:10.1038/s41467-024-46540-0)
Supplement: Supplementary file 4 — Supplementary Data 1 [file 41467_2024_46540_MOESM4_ESM.pdf]

Supplementary Data 1

Subject ID	Clinical diagnosis	scRNA-seq	IF	PCR	Biopsy location	Age Range (years)	Sex	Race	Disease duration of underlying AD	Ongoing systemic treatment	Previous treatments	Ongoing topical treatment***	IGA**	Total serum IgE*
112 – HC1	Healthy control skin	X			Trunk	50-60	F	White	n.a.	None	n.a.	n.a.	n.a.	<100
115 – HC2	Healthy control skin	X			Trunk	40-50	M	White	n.a.	None	n.a.	n.a.	n.a.	<100
116 – HC3	Healthy control skin	X			Trunk	50-60	F	White	n.a.	None	n.a.	n.a.	n.a.	<100
121 – HC4	Healthy control skin	X			Trunk	40-50	F	White	n.a.	None	n.a.	n.a.	n.a.	<100
74 – Trunk AD1	Atopic dermatitis	X			Trunk	20-30	M	White	24 years	None	TCS, TCI	Moisturizer	3	351
75 – Trunk AD2	Atopic dermatitis	X			Trunk	20-30	F	White	10 years	None	TCS, Phototherapy (NB-UVB)	Moisturizer/ homeopathic ointments	4	8
77 – Trunk AD3	Atopic dermatitis	X			Trunk	20-30	M	White	12 years	None	TCS, Phototherapy (NB-UVB)	Moisturizer	3	2045
81 – Trunk AD4	Atopic dermatitis	X			Trunk	50-60	M	White	50 years	None	TCS, TCI, Phototherapy (NB-UVB)	Moisturizer	4	>5000
96 – Trunk AD5	Atopic dermatitis	X			Trunk	50-60	M	White	Since childhood	None	TCS	None	4	>5000
120 – DAHND1	Dupilumab-associated head neck dermatitis	X			Neck	40-50	M	White	Adult-onset	Dupilumab 300mg eow	Systemic corticosteroids, TCS	Moisturizer, TCI	1	82.8
126 – DAHND2	Dupilumab-associated head neck dermatitis	X			Neck	40-50	M	White	Since childhood	Dupilumab 300mg eow	UV, TCS	Moisturizer	1	2874
133 – DAHND3	Dupilumab-associated head neck dermatitis	X			Neck	40-50	M	White	Since childhood	Dupilumab 300mg eow	PUVA, TCS	Moisturizer	1	>5000
137 – DAHND4	Dupilumab-associated head neck dermatitis	X			Neck	30-40	M	White	Since childhood	Dupilumab 300mg eow	UVB, TCS	Moisturizer	1	2247
147 – DAHND5	Dupilumab-associated head neck dermatitis	X			Neck	50-50	M	White	Since childhood	Dupilumab 300mg eow	MTX, PUVA, TCS, UVB	None	2	>5000
161 – DAHND6	Dupilumab-associated head neck dermatitis	X			Neck	30-40	F	White	Since childhood	Dupilumab 300mg eow	UVB, systemic corticosteroids	Topical erythromycin, moisturizers	1	1648
148 – Head/neck AD1	Atopic dermatitis	X			Neck	20-30	M	White	Since childhood	None	TCS, TCI	Moisturizer	3	2650
151 – Head/neck AD2	Atopic dermatitis	X			Neck	50-60	M	White	Since childhood	None	TCS	Moisturizer	2	3803
176 – Head/neck AD3	Atopic dermatitis	X			Neck	80-90	M	White	Adult-onset	None	TCS, systemic corticosteroids	None	4	111

177 – Head/neck AD4	Atopic dermatitis	X			Neck	40-50	M	White	Since childhood	None	TCS, TCI	Moisturizer	3	2349
180 – Head/neck AD5	Atopic dermatitis	X			Neck	50-60	M	White	Adult-onset	None	TCS	None	4	991
HC5	Healthy control skin		X	X	Trunk	40-50	F	White	n.a.	None	n.a.	n.a.	n.a.	<100
HC6	Healthy control skin		X	X	Trunk	50-60	M	White	n.a.	None	n.a.	n.a.	n.a.	<100
HC7	Healthy control skin		X	X	Trunk	30-40	F	White	n.a.	None	n.a.	n.a.	n.a.	<100
HC8	Healthy control skin			X	Trunk	30-40	F	White	n.a.	None	n.a.	n.a.	n.a.	<100
HC9	Healthy control skin			X	Trunk	40-50	F	White	n.a.	None	n.a.	n.a.	n.a.	<100
HC10	Healthy control skin			X	Trunk	50-60	F	White	n.a.	None	n.a.	n.a.	n.a.	<100
HC11	Healthy control skin			X	Trunk	40-50	M	White	n.a.	None	n.a.	n.a.	n.a.	<100
HC12	Healthy control skin			X	Trunk	n.a.	M	White	n.a.	None	n.a.	n.a.	n.a.	<100
HC13	Healthy control skin			X	Trunk	40-50	M	White	n.a.	None	n.a.	n.a.	n.a.	<100
Trunk AD6	Atopic dermatitis		X	X	Trunk	70-80	M	White	Adult-onset	None	TCS	None	3	64.7
Trunk AD7	Atopic dermatitis		X	X	Trunk	20-30	M	White	Since childhood	None	TCS	Moisturizer	4	>5000
Trunk AD8	Atopic dermatitis		X	X	Trunk	60-70	M	White	Since childhood	None	TCS	Moisturizer, TCS	3	>5000
Trunk AD9	Atopic dermatitis		X	X	Trunk	20-30	M	White	Adult-onset	None	TCS	Moisturizer	3	582
Head/Neck AD6	Atopic dermatitis		X	X	Neck	50-60	F	White	Since childhood	None	TCS	None	3	1468
Head/Neck AD7	Atopic dermatitis		X	X	Neck	30-40	M	White	Since childhood	None	PUVA, TCS	Moisturizer	4	253
Head/Neck AD8	Atopic dermatitis		X	X	Neck	50-60	M	White	Adult-onset	None	PUVA, TCS, systemic corticosteroids	Moisturizer, TCS 1/week	3	4681
Head/Neck AD9	Atopic dermatitis		X	X	Neck	50-60	M	Asian	Since childhood	None	MTX, systemic corticosteroids, TCS	None	4	>5000
Head/Neck AD10	Atopic dermatitis		X	X	Neck	20-30	M	White	Since childhood	None	TCS, Dupilumab	Moisturizer, TCI 1-2/week	4	40.2
DAHND7	Dupilumab-associated head neck dermatitis		X	X	Neck	40-50	F	White	Since childhood	Dupilumab 300mg eow	UVB, TCS	Moisturizer	1	28.8
DAHND8	Dupilumab-associated head neck dermatitis		X	X	Neck	30-40	M	White	Since childhood	Dupilumab 300mg eow	MTX, CsA, IVIG, UVB, PUVA	Moisturizer	0	>5000
DAHND9	Dupilumab-associated head neck dermatitis		X	X	Neck	40-50	F	White	Since childhood	Dupilumab 300mg eow	CsA, TCS, systemic corticosteroids	Moisturizer	1	>5000

Treated trunk AD1	Trunk AD dupilumab-treated			X	Trunk	50-60	F	White	Since childhood	Dupilumab 300mg eow	TCS, UVB, PUVA	Moisturizer	0	1204
Treated trunk AD2	Trunk AD dupilumab-treated			X	Trunk	30-40	M	White	Since childhood	Dupilumab 300mg eow	TCS	Moisturizer, plant-derived oils	1	>5000
Treated trunk AD3	Trunk AD dupilumab-treated			X	Trunk	40-50	F	White	Since childhood	Dupilumab 300mg eow	CsA, TCS, systemic corticosteroids	Moisturizer	1-2	>5000
Treated trunk AD4	Trunk AD dupilumab-treated			X	Trunk	50--60	M	White	Since childhood	Dupilumab 300mg eow	UVB, MTX, TCS	Moisturizer	1	3862
Treated trunk AD5	Trunk AD dupilumab-treated			X	Trunk	40-50	M	White	Since childhood	Dupilumab 300mg eow, valaciclovir prophylaxis	UVB, TCS	Moisturizer	2	1716
