# Supplementary material for: Dupilumab-associated head and neck dermatitis shows a pronounced type 22 immune signature mediated by oligoclonally expanded T cells
Source: Nat Commun. 2024 Apr 2;15:2839. doi: 10.1038/s41467-024-46540-0 (PMC10987549; doi:10.1038/s41467-024-46540-0)
Supplement: Supplementary file 13 — Reporting Summary [file 41467_2024_46540_MOESM13_ESM.pdf]

nature portfolio

Corresponding author(s): Patrick Brunner

Last updated by author(s): Feb 1, 2024

Reporting Summary

Nature Portfolio wishes to improve the reproducibility of the work that we publish. This form provides structure for consistency and transparency in reporting. For further information on Nature Portfolio policies, see our Editorial Policies and the Editorial Policy Checklist.

Statistics

For all statistical analyses, confirm that the following items are present in the figure legend, table legend, main text, or Methods section.

n/a	Confirmed	
☐	☒	The exact sample size (*n*) for each experimental group/condition, given as a discrete number and unit of measurement
☐	☒	A statement on whether measurements were taken from distinct samples or whether the same sample was measured repeatedly
☐	☒	The statistical test(s) used AND whether they are one- or two-sided<br>*Only common tests should be described solely by name; describe more complex techniques in the Methods section.*
☐	☒	A description of all covariates tested
☐	☒	A description of any assumptions or corrections, such as tests of normality and adjustment for multiple comparisons
☐	☒	A full description of the statistical parameters including central tendency (e.g. means) or other basic estimates (e.g. regression coefficient) AND variation (e.g. standard deviation) or associated estimates of uncertainty (e.g. confidence intervals)
☐	☒	For null hypothesis testing, the test statistic (e.g. *F*, *t*, *r*) with confidence intervals, effect sizes, degrees of freedom and *P* value noted<br>*Give P values as exact values whenever suitable.*
☒	☐	For Bayesian analysis, information on the choice of priors and Markov chain Monte Carlo settings
☒	☐	For hierarchical and complex designs, identification of the appropriate level for tests and full reporting of outcomes
☒	☐	Estimates of effect sizes (e.g. Cohen's *d*, Pearson's *r*), indicating how they were calculated

Our web collection on statistics for biologists contains articles on many of the points above.

Software and code

Policy information about availability of computer code

Data collection	10x Genomics (version 6.1.2)
Data analysis	All data was processed using open source software only. The respective tools are CellRanger from 10x Genomics (version 6.1.2), Seurat (v. 4.0.4 - 4.4.0), Scran (v. 1.26.2), Bioconductor (v. 3.16), BiocManager (v. 1.30.19),SingleCellExperiment version (1.20.1) EnhancedVolcano version (1.16.0) and scDblFinder (v. 1.10.0).The processed 10X Genomics datasets generated in this study have been deposited in the Gene Expression Omnibus (GEO) database under accession code GSE230575 [https://www.ncbi.nlm.nih.gov/geo/query/acc.cgi?acc=GSE230575]. Data from healthy control and trunk AD samples are equivalent to samples with the GEO accession numbers GSE173205 [https://www.ncbi.nlm.nih.gov/geo/query/acc.cgi?acc=GSE173205] and GSE222840 [https://www.ncbi.nlm.nih.gov/geo/query/acc.cgi], respectively, from previously published datasets from our laboratory. The code for the data generated in this study is provided in the Supplementary folder "Supplementary Software".

For manuscripts utilizing custom algorithms or software that are central to the research but not yet described in published literature, software must be made available to editors and reviewers. We strongly encourage code deposition in a community repository (e.g. GitHub). See the Nature Portfolio guidelines for submitting code & software for further information.

Data

Policy information about availability of data

All manuscripts must include a data availability statement. This statement should provide the following information, where applicable:

- Accession codes, unique identifiers, or web links for publicly available datasets
- A description of any restrictions on data availability
- For clinical datasets or third party data, please ensure that the statement adheres to our policy

The processed 10X Genomics datasets generated in this study have been deposited in the Gene Expression Omnibus (GEO) database under accession code GSE230575 [https://www.ncbi.nlm.nih.gov/geo/query/acc.cgi?acc=GSE230575]. Data will be made publicly available upon acceptance of the manuscript.
Data from healthy control and trunk AD samples are equivalent to samples with the GEO accession numbers GSE173205 [https://www.ncbi.nlm.nih.gov/geo/query/acc.cgi?acc=GSE173205] and GSE222840 [https://www.ncbi.nlm.nih.gov/geo/query/acc.cgi], respectively.
Source data are provided with this paper.

Research involving human participants, their data, or biological material

Policy information about studies with human participants or human data. See also policy information about sex, gender (identity/presentation), and sexual orientation and race, ethnicity and racism.

Reporting on sex and gender	Sex (self reported) is included in Table 1 of the manuscript
Reporting on race, ethnicity, or other socially relevant groupings	Race (self reported) is included in Table 1 of the manuscript
Population characteristics	Population characteristics are given in Table 1
Recruitment	Participants were recruited on a first-come-first-serve basis via the outpatient clinic of the Department of Dermatology, Medical University of Vienna, Austria. Every patient meeting inclusion/exclusion criteria and willing to participate was included.
Ethics oversight	The study was approved by the Ethics Committee of the Medical University of Vienna, Austria (EK 1360/2018).

Note that full information on the approval of the study protocol must also be provided in the manuscript.

Field-specific reporting

Please select the one below that is the best fit for your research. If you are not sure, read the appropriate sections before making your selection.

☒ Life sciences ☐ Behavioural & social sciences ☐ Ecological, evolutionary & environmental sciences

For a reference copy of the document with all sections, see nature.com/documents/nr-reporting-summary-flat.pdf

Life sciences study design

All studies must disclose on these points even when the disclosure is negative.

Sample size	Sample size was a result of feasibility (skin biopsies from sensitive areas such as the head/neck area) and experience from previous single-cell RNA sequencing studies (Ref. PMID 34583709, PMID 33483337).
Data exclusions	No data was excluded
Replication	Single-cell RNA sequencing data were analyzed in its entirety once all patients were recruited. Data obtained were then confirmed in independent sample sets of skin biopsies using immunofluorescence stainings as well as quantitative RT-PCR. These experiments were performed in a single run, independently from each other.
Randomization	Not applicable, patients received treatment as standard of care. Given small sample size, patients were recruited in a first come first serve basis.
Blinding	No blinding was performed in this study as patients received treatment as standard of care by the treating physician who also included these patients in the study.

Reporting for specific materials, systems and methods

We require information from authors about some types of materials, experimental systems and methods used in many studies. Here, indicate whether each material, system or method listed is relevant to your study. If you are not sure if a list item applies to your research, read the appropriate section before selecting a response.

Materials & experimental systems

n/a	Involved in the study
☐	☒ Antibodies
☒	☐ Eukaryotic cell lines
☒	☐ Palaeontology and archaeology
☒	☐ Animals and other organisms
☐	☒ Clinical data
☒	☐ Dual use research of concern
☒	☐ Plants

Methods

n/a	Involved in the study
☒	☐ ChIP-seq
☒	☐ Flow cytometry
☒	☐ MRI-based neuroimaging

Antibodies

Antibodies used

AF647-conjugated anti-CD79a (1:50, clone: HM47, Cat: 333516, Lot: B313857, Biolegend), PE/Dazzle 594-conjugated anti-CD207 (Langerin) (1:20, clone: 4C7, Cat: 144212, Lot: B362308, Biolegend), AF647-conjugated anti-IL-22BP (1:20, clone: # 875504, Cat: FAB10871R-100UG, Lot: 1713824, R&D Systems), PE-conjugated anti-CD129 (IL-9R) (1:30, clone: AH9R7, Cat: 310404, Lot: B340453, Biolegend), AF488-conjugated anti-CD8 (1:10, clone: SK1, Cat: 344716, Lot: B351669, Biolegend), AF647-conjugated anti-CD314 (KLRK1) (1:20, clone: 1D11, Cat: 320826, Lot: B360539, Biolegend), AF647-conjugated anti-IL22 (1:20, clone: MH22B2, Cat: 567160, Lot: 3101605, BD Biosciences), AF546-conjugated goat-anti-mouse IgG (1:400, Cat: A11030, Lot: 2026145, Thermo Fisher Scientific).

Validation

Validation statements regarding the tested applications of Biolegend antibodies can be found by their respective Lot numbers in the Certificate of Analysis section web-page [https://www.biolegend.com/de-at/global-elements/certificate-of-analysis]. Additionally, corresponding isotype controls, such as AF647-conjugated mouse IgG1 (1:10, MOPC-21, Cat: 400136, Lot: B287199, Biolegend), AF488-conjugated mouse IgG1 (1:20, MOPC-21, Cat: 400129, Lot: B354284, Biolegend), PE-conjugated mouse IgG2b (1:10, MG2b-57, Cat: 401208, Lot: B353563, Biolegend), AF647-conjugated mouse IgG2a (1:2.5, MOPC-173, Cat: 400234, Lot: B356813, Biolegend), AF594-conjugated mouse IgG2a (1:50, MOPC-173, Cat: 400280, Lot: B331958, Biolegend), and AF647-conjugated mouse IgG2b (1:50, MPC-11, Cat: 400330, Lot: B379318, Biolegend), were used to confirm staining specificity.

Clinical data

Policy information about clinical studies
All manuscripts should comply with the ICMJEguidelines for publication of clinical research and a completedCONSORT checklist must be included with all submissions.

Clinical trial registration

not applicable, this is not a clinical trial, patients received therapy as standard of care

Study protocol

Approved by the Ethics Committe of the Medical University of Vienna, Austria.

Data collection

by clinical investigator / treating physician during standard of care patient visits

Outcomes

not applicable, this is not a clinical trial, patients received treatment as standard of care.
